# Integrative genomics analysis of eQTL and GWAS summary data identifies *PPP1CB* as a novel bone mineral density risk genes

**DOI:** 10.1042/BSR20193185

**Published:** 2020-04-21

**Authors:** Yu Zhai, Lu Yu, Yang Shao, Jianwei Wang

**Affiliations:** 1 Changzhou Hospital Affiliated to Nanjing University of Chinese Medicine, Changzhou 213003, Jiangsu, China; 2 Wuxi Hospital Affiliated to Nanjing University of Chinese Medicine, Wuxi 214071, Jiangsu, China

**Keywords:** BMD, gene expression, genetic variants, GWAS, osteoporosis, susceptible genes

## Abstract

In recent years, multiple genome-wide association studies (GWAS) have identified numerous susceptibility variants and risk genes that demonstrate significant associations with bone mineral density (BMD). However, exploring how these genetic variants contribute risk to BMD remains a major challenge. We systematically integrated two independent expression quantitative trait loci (eQTL) data (*N* = 1890) and GWAS summary statistical data of BMD (*N* = 142,487) using *Sherlock* integrative analysis to reveal whether expression-associated variants confer risk to BMD. By using *Sherlock* integrative analysis and MAGMA gene-based analysis, we found there existed 36 promising genes, for example, *PPP1CB, XBP1*, and *FDFT1*, whose expression alterations may contribute susceptibility to BMD. Through a protein–protein interaction (PPI) network analysis, we further prioritized the *PPP1CB* as a hub gene that has interactions with predicted genes and BMD-associated genes. Two eSNPs of rs9309664 (*P*_eQTL_ = 1.42 × 10^−17^ and *P*_GWAS_ = 1.40 × 10^−11^) and rs7475 (*P*_eQTL_ = 2.10 × 10^−6^ and *P*_GWAS_ = 1.70 × 10^−7^) in *PPP1CB* were identified to be significantly associated with BMD risk. Consistently, differential gene expression analysis found that the *PPP1CB* gene showed significantly higher expression in low BMD samples than that in high BMD samples based on two independent expression datasets (*P* = 0.0026 and *P* = 0.043, respectively). Together, we provide a convergent line of evidence to support that the *PPP1CB* gene involves in the etiology of osteoporosis.

## Introduction

Osteoporosis is a common age-related complex disease. The disease leads to a huge economic burden on health care systems with a cost of about $17 billion per year in the United States, and it is expected to be $25.5 billion annually by the year of 2025 [1]. Bone mineral density (BMD) is the most commonly used indicator to assess the increased risk of a fracture that is the characteristics of osteoporosis [2]. BMD is highly influenced by genetic factors, and the narrow-sense heritability of BMD has been estimated to be approximately 85% [3–5]. Consequently, there exists a considerable interest in identifying the genetic basis of osteoporosis for developing effective methods for its treatment and prevention.

Genome-wide association study (GWAS) is a good approach that is capable of simultaneously examining the genetic links between millions of variants and phenotypes of interest. In the recent decade, numerous GWASs of osteoporosis and BMD have successfully reported multiple genetic variants and related susceptible genes that show robust associations [4,6–10]. These identified genetic loci from GWAS contain a wealth of information with the potential to explore new risk genes and molecular pathways that have critical roles in bone biology and inform drug discovery [6,11]. Nevertheless, it remains elusive on how these genetic loci confer risk to osteoporosis and BMD. Additionally, many genetic loci with weak or modest effects were hard to be detected in a single GWAS study due to their genome-wide threshold of strict statistical significance. Thus, more studies are warranted to uncover the underlying effects of the small-to-modest GWAS association signals on BMD that may be conducive to understand the missing heritability of this trait.

Although more BMD-associated genetic loci warrant to be identified, growing evidence has strongly demonstrated that the altered expression of gene has a crucial part in the pathogenesis of osteoporosis [12–14]. Furthermore, recent studies [15–18] have employed systematically integrative approaches to integrate the GWAS summary data and expression quantitative trait loci (eQTL) data to explore the potent regulatory effects of the risk variants in reported GWAS. He et al. [15] introduced a Bayesian statistical approach of *Sherlock* to systematically reveal the cis- and trans-regulatory effects of risk genes on complex diseases based on GWAS summary data and eQTL data. By using this bioinformatics tool, numerous studies have identified many novel risk genes, which cannot be found with the use of the GWAS approach alone, for different complex traits, such as schizophrenia [19], gout disease [20], and major depressive disorders [21,22].

The primary goal of the current investigation is to explore whether genetic variants linked with gene expression confer risk to BMD and identify BMD-associated genes based on combining both GWAS and eQTL data by using a Bayesian method of *Sherlock*. To further validate the potent roles of these identified BMD-associated genes in osteoporosis etiology, we re-performed the integrative analysis in an independent eQTL dataset and applied additional genomic analyses based on RNA expression data.

## Materials and methods

### Bone mineral density GWAS data

We employed a large-scale GWAS summary statistics data on BMD [10] for searching risk genetic variants. Briefly, this BMD-associated GWAS study was reported recently by the Genetic Factors for Osteoporosis (GEFOS) Consortium, which comprises 142,487 individuals in total. All individuals have signed the informed consent and the ethical approval was obtained from the Northwest Multi-centre Research Ethics Committee. Data were imputed centrally based on the UK10K/1000G combined imputation panel (hg19). Only SNPs down to a MAF of 0.1% and with an info-score threshold of >0.4 were included for analysis. The association information of genetic variants in GWAS including the name of each SNP and related *P* value was employed as input in *Sherlock* analysis and MAGMA analysis. There were a total of 17,166,350 SNPs in the chosen GWAS employed as input in the current investigation. For more detailed information, please refer to the original published article [10] and the GEFOS website (http://www.gefos.org/).

### Discovery eQTL data

We used the monocyte eQTL data that were from a single-center cohort study of the Gutenberg Heart Study (GHS) as discovery eQTL, including a number of 1490 unrelated study participants with both DNA and RNA available. Informed consent was signed from all subjects. The *Affymetrix* Genome-Wide Human SNP Array 6.0 (http://www.affymetrix.com) was applied to conduct the genotyping for each sample. Based on the standard criteria of quality control for SNPs in the Affymetrix SNP Array 6.0 including the *P* value for Hardy–Weinberg equilibrium >1 × 10^−4^, the calling rate of genotype >98%, and the frequency of minor allele > 0.01, we removed 225,042 low-quality SNPs and obtained 675,350 SNPs for further analysis. With the use of the *Illumina* HT-12 v3 BeadChip (http://www.Illumina.com), genome-wide expression analysis was conducted based on RNA samples from monocytes. There were a total of 37,804 genes included in the *Illumina* HT-12 BeadChip. Of these genes, a number of 22,305 genes were treated as being prominently expressed. We removed 8058 not well-characterized genes and used 12,808 well-characterized genes to carry out a subsequent eQTL analysis. For more information, please refer to the original published article [23].

### Independent validation eQTL data

We also employed an independent eQTL dataset published by Dixon and coworkers [24], which have created a global map of the effects of polymorphism on gene expression. Ethical approval was given by the Multicentre Research Ethics Committees (U.K.), and written informed consent was given by all participants. Whole-genome genotyping was conducted according to manufacturers’ instructions using the Human Hap300 Genotyping BeadChip (Illumina) and the Sentrix Human-1 Genotyping BeadChip in a BeadChip with full automation. Genome-wide expression analysis was carried out on lymphoblastoid RNA samples using the U133 Plus 2.0 GeneChips (Affymetrix), according to the manufacturers’ protocol. After strict quality control, genotypes and gene expression of 400 participants were used to generate eQTL resources. Please refer to the original paper for more detailed information [24].

### Sherlock integrative analysis using discovery eQTL data

Given the vast majority of the GWAS-identified variants associated with traits of interest are located in non-coding genomic regions [25], it is likely to infer that the identified susceptibility variants influence the expression level of the relevant gene rather than the function of its protein. Based on the putative assumption that specific gene expression may convey susceptibility to BMD, we employed the approach of *Sherlock* integrative analysis to integrate GWAS summary statistics on BMD from Kemp et al. [10] and circulating monocyte eQTL data from Zeller et al. [23]. The statistical inference procedures of the *Sherlock* method are described as following: *Sherlock* first searches expression-associated SNPs (as called eSNPs) in the human monocyte samples with the use of the eQTL data from the study of Zeller et al. [23]. Second, *Sherlock* tool assesses the potent association of eSNPs with BMD using the GWAS summary data from Kemp et al. [10]. There exists three scenarios: (1) A positive score would be recorded based on a specific eSNP of a gene in the chosen GWAS shows a significant association with BMD; (2) A negative score would be assigned based on a specific eSNP of this gene shows a non-significant association with BMD; (3) No score would be assigned based on an SNP was not eSNP but shows a significant association with BMD. The summed score of a specific gene was depended on the number of SNPs with the evidence combined from both GWAS and eQTL data. Concerning each gene, *Sherlock* software conducted a Bayesian statistical inference with the use of the integrated data of the potent eSNPs of the gene to examine whether the alteration in the expression of the specific gene has any influence on BMD risk. Through computing the logarithm of Bayes factor, *Sherlock* tool predicts BMD-associated genes by combining the data from eQTL and GWAS summary statistics. With a comparison of existing traditional analysis, which commonly neglects moderate SNPs, *Sherlock* tool based on an effective Bayesian model employs SNPs in GWAS with moderately and strongly genetic association signals. For *Sherlock* analysis, the method of Bonferroni correction was applied to correct the *P*-values of genes.

### Sherlock integrative analysis using independent eQTL data

Subsequently, to replicate whether these identified genes are genuine BMD-associated risk genes, we conducted a further *Sherlock* integrative analysis based on an independent eQTL data (biological validation), which were reported by a study of Dixon and his colleagues [24]. All the parameter settings of *Sherlock* analysis were the same with that of discovery eQTL data. Consistently, the *P*-value of each gene was corrected by utilized the Bonferroni correction method.

### MAGMA gene-based analysis

We used the software of Multi-marker Analysis of GenoMic Annotation (MAGMA) [26] as an independent bioinformatics tool to perform a gene-based enrichment analysis for technically validate the identified genes from *Sherlock* Bayesian analysis. For the MAGMA tool, we could extract the name of each SNP with its *P*-value from BMD GWAS summary statistics as input to identify the significant association signals at a gene level. SNPs mapped into a specific gene or the genomic region extended ±20 kb downstream or upstream of the gene were chosen to identify multiple variants convergent effects and collectively calculate the *P*-value of the gene risk to BMD. The linkage disequilibrium (LD) information between chosen SNPs was computed based on the 1000 Genome European panel. The Bonferroni correction method was used for multiple testing.

### Null GWAS data

To make sure these identified BMD-associated genes because of genetic biology instead of random events, we carried out a MAGMA gene-based analysis with the same parameters by using an artificial GWAS dataset (as a negative control dataset). The control GWAS was derived from a real genetic dataset with a number of 3960 participants published by Landi and colleagues [27]. We randomly assigned the phenotype of BMD to each participant to construct a Null trait. In view of the Null GWAS was supposed to be no true genetic effect, the power of this analysis based on relatively small sample size is not an issue.

### Pathway enrichment analysis

To reveal the biological interpretation of these prioritized BMD-associated genes from the discovery eQTL data, we used the ClueGO [28], an easy use Cytoscape plug-in [29], to create a functionally organized pathway term network. We used gene-set data from the two most recent sources of KEGG [30] and GO [31] (including biological process, molecular function, and cellular component), ensuring an up-to-date functional analysis. We also employed the method of “*GO Term Fusion”*, which fusion of GO parent-child terms based on similar associated genes, to reduce the redundancies of GO terms. Based on the two-sided test of hypergeometric distribution, the *P* values were calculated by depletion or enrichment for groups and terms. The Bonferroni step down correction was applied to correct *P* values for multiple testing.

### Computer-based permutation analysis

To reveal whether *Sherlock*-identified risk genes in the discovery stage (corrected *P* < 0.05: *N* = 147 genes; raw *P* < 0.05: *N* = 2064 genes) were significantly overlapped with genes identified from Sherlock validation analysis (corrected *P* < 0.05: *N* = 98 genes; raw *P* < 0.05: *N* = 2031 genes) or MAGMA analysis (corrected *P* < 0.05: *N* = 1106 genes), respectively, we conducted a computer-based permutation analysis [32]. For this permutation analysis, we randomly selected the number of genes as same as significantly identified genes from background genes for 100,000 times and documented the overlapped rate with genes from the *Sherlock* discovery stage. Then, we counted how many times the counts of overlapped genes were larger than the number detected from real data 100,000 times. The probability of the observed number considered as the empirical *P* value.

### Protein–protein interaction (PPI) network

Multiple lines of evidence have documented that genes associated with complicated disorders are more tend to be interacted [14,33,34]. Thus, PPI network-based analysis has been extensively applied to search for groups of functionally related genes that may collectively contribute risk to complex disease. We conducted a PPI network-based analysis of these identified BMD-associated risk genes based on the GeneMANIA database (http://www.genemania.org) [35]. The GeneMANIA is a flexible and user-friendly tool for analyzing a list of genes to infer the functions of inputted genes and narrowing down the number of genes for molecular experiments. If inputting a query list of genes, the GeneMANIA tool extends the inputted genes with functionally similar genes based on current available proteomics and genomics data.

### Identification of BMD-associated genes expression profiles

*Sherlock* analysis identifies the disease-associated gene based on the assumption that risk gene abnormal expression may implicate in the pathogenesis of disease of interest. To reveal whether the identified susceptibility genes are differentially expressed in low BMD compared with high BMD group, we obtained two existing RNA expression datasets available in the NCBI’s GEO database (Accession Nos. GES2208 and GSE7429). GSE2208 contains transcriptome data of 10 low BMD subjects and 10 high BMD subjects. GSE7429 contains RNA expression data of 9 low BMD subjects and 10 high BMD subjects. For more detailed information on the sample collections and other procedures of quality control, please find two original articles [24,36]. The R package of *corrplot* was utilized to show the differential co-expression patterns of these identified risk genes between high BMD and low BMD group. The difference between high BMD and low BMD groups was compared for significance with the Student’s *T*-test. A *P* value < 0.05 was considered statistically significant.

## Results

### Prioritization of BMD-associated risk genes

**Figure 1:**
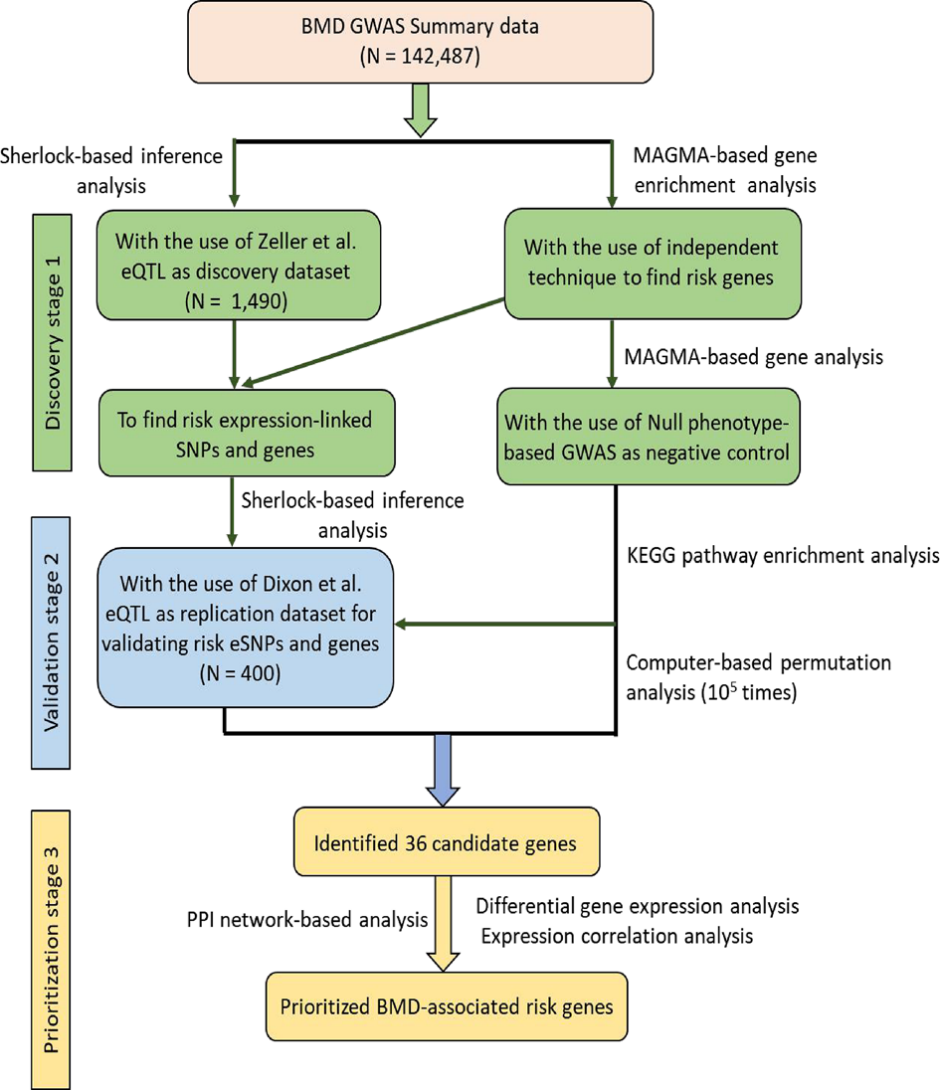
The workflow of prioritizing the BMD-associated genes

The workflow of the present study design is shown in Figure 1. To determine whether these genes with abnormal expression may convey risk to BMD, we applied the *Sherlock* tool to integrate GWAS summary data with a total of 17,166,350 SNPs (*N* = 142,487 samples) with eQTL data based on 1490 samples. The *Sherlock* integrative analysis, which is often used to find disease-associated genes by matching patterns of GWAS and eQTL, was carried out for the Bayesian statistical inference. Based on this Bayesian method, we identified 147 top BMD-associated genes whose abnormal expression may influence BMD risk at the threshold of *P* < 1.57×10^−6^ (Bonferroni corrected *P* < 0.05; Supplemental Table S1) and 2064 suggestive BMD-associated genes (*P* < 0.05). For each of these identified significant genes, for example, *NR1H3, MVK, ACP2, EPB41L2, ZEP57, PBX1*, and *PPP1CB*, at least one SNP showed significant association with the expression of this gene and BMD risk simultaneously, inferring that these SNPs are promising functional candidates with potential cis- and/or trans-regulatory effects on risk genes. Furthermore, we performed a pathway-based analysis for these 147 significant genes and there were 11 pathways showing significant enrichment (Table 1); such as the pathway of negative regulation of type 2 immune response (*P* = 0.00018), inactivation of MAPK activity (*P* = 0.0011), and PPAR signaling pathway (*P* = 0.0021).

**Table 1:** Significant pathways enriched by identified BMD-associated genes based on discovery dataset

Pathway ID	Pathway terms	Associated genes proportion	*P* value	Corrected *P* value	Associated genes
GO:0002829	Negative regulation of type 2 immune response	0.200	0.00018	0.0018	*ARG2, SOCS5, STAT6*
GO:1904294	Positive regulation of ERAD pathway	0.200	0.00018	0.0018	*ATXN3, EDEM2, XBP1*
GO:0071498	Cellular response to fluid shear stress	0.143	0.00050	0.0045	*KLF2, SOCS5, XBP1*
GO:0000188	Inactivation of MAPK activity	0.111	0.00106	0.0085	*DUSP1, DUSP12, DUSP3*
GO:1901800	Positive regulation of proteasomal protein catabolic process	0.047	0.00122	0.0085	*ATXN3, DAB2, EDEM2, SOCS5, XBP1*
KEGG:03320	PPAR signaling pathway	0.056	0.00210	0.0126	*CPT1A, FABP7, FADS2, NR1H3*
KEGG:04142	Lysosome	0.041	0.00235	0.0118	*ACP2, AP3D1, CTSB, GUSB, IDUA*
GO:0048246	Macrophage chemotaxis	0.079	0.00287	0.0115	*LGALS3, MMP28, S100A8*
GO:1904375	Regulation of protein localization to cell periphery	0.043	0.00529	0.0159	*CIB1, DAB2, EPB41L2, SPTBN1*
GO:0030170	Pyridoxal phosphate binding	0.050	0.01034	0.0207	*GCAT, KYAT3, PYGM*
GO:1901880	Negative regulation of protein depolymerization	0.048	0.01131	0.0113	*CIB1, SPTBN1, TRIOBP*

### Validation of BMD-associated risk genes

To further validate our results in the discovery stage, we replicated the findings of *Sherlock* integrative analysis with the use of an independent of Dixon et al. eQTL dataset (Replication dataset: *N* = 400). By using the same parameter settings, *Sherlock* integrative analysis based on independent eQTL data identified 98 significant genes (Supplementary Table S3; Bonferroni corrected *P* < 0.05) and 2031 suggestive genes (*P* < 0.05). Among these 147 significant genes identified from the discovery stage, there were 48 genes validated in the replication stage (*P* < 0.05). In both the discovery and validation stage, there were 11 BMD-associated genes reached Bonferroni correction significance, namely *CPT1A, CKB, MRPL21, ZFP57, ACP2, PPP1CB, ST7L, EPB41L2, PBX1, AMT*, and *VNN3* (corrected *P* values < 0.05; Supplementary Figure S1). Multiple cis-SNPs in these identified genes convey risk to BMD (Supplementary Tables S2 and S4).

### MAGMA-based gene enrichment analysis of BMD

**Figure 2:**
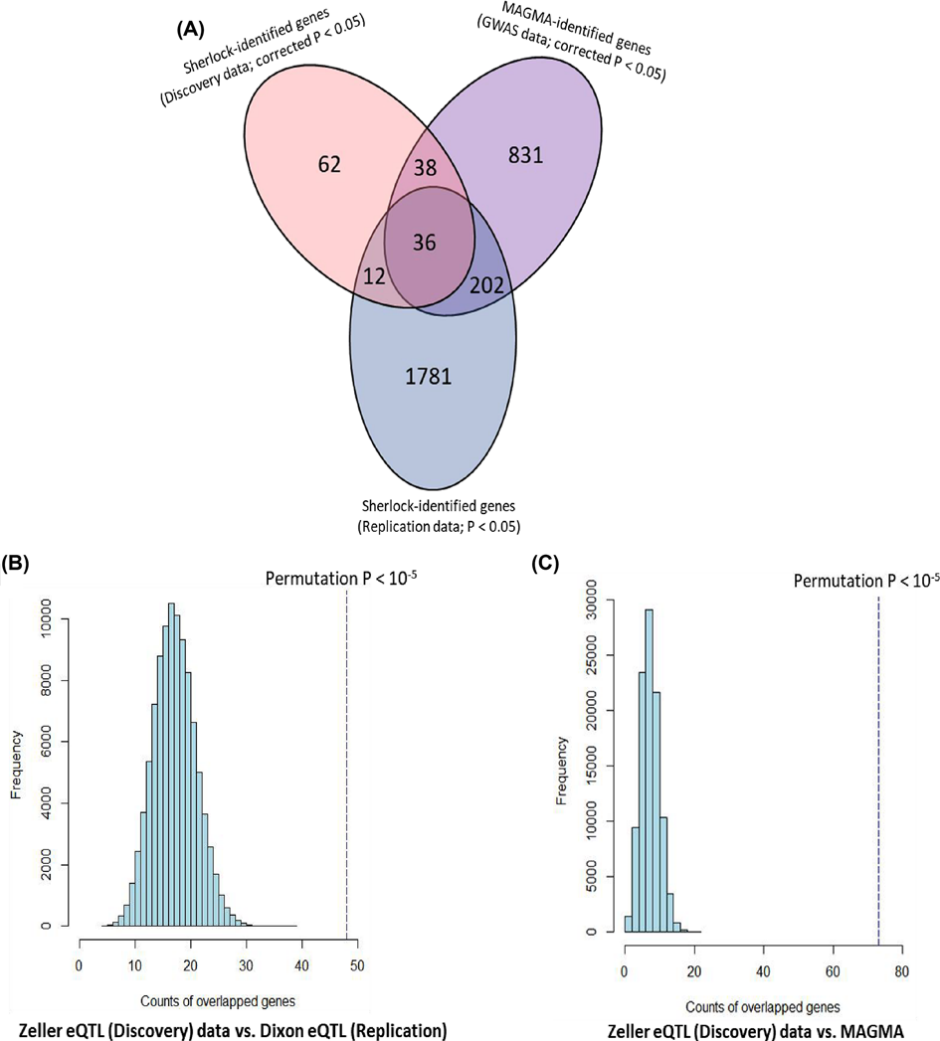
Convergent evidence of BMD-associated genes from independent datasets (**A**) Venn plot of BMD-relevant genes based on three datasets: *Sherlock*-identified genes from the discovery dataset (corrected *P*<0.05); *Sherlock*-identified genes from the validation dataset (*P*<0.05); MAGMA-identified genes from the GWAS dataset (corrected *P*<0.05). (**B**) Computer-based permutation analysis (100,000 times) of the counts of risk genes from Zeller eQTL dataset (corrected *P*<0.05; discovery stage) overlapped with that from Dixon eQTL dataset (*P*<0.05; Replication stage). (**C**) Computer-based permutation analysis (100,000 times) of the counts of risk genes from Zeller eQTL dataset (corrected *P*<0.05; discovery stage) overlapped with that from MAGMA-based dataset (corrected *P*<0.05; technical validation stage).

To ensure the reliability of these identified BMD-risk genes, we carried out an independent technical approach of MAGMA gene-based analysis. We found a number of 1106 genes were significantly associated with the phenotype of BMD based on Bonferroni correction for multiple testing (corrected *P* < 0.05). The top-ranked association signals were of *CPED1* (corrected *P* = 7.98 × 10^−202^), *CCDC170* (corrected *P* = 1.78 × 10^−182^), and *WNT16* (corrected *P* = 3.96 × 10^−137^). There were 36 significant genes from MAGMA analysis overlapped with genes identified from *Sherlock* analysis (correlated *P* < 0.05 of genes from the discovery stage; Figure 2A and Table 2). We observed that none of these 36 identified genes show significant associations in the MAGMA analysis of Null GWAS (Table 2). In addition, a number of 86 MAGMA-identified significant genes were overlapped with *Sherlock*-identified genes (*P* < 0.05 of genes from the discovery stage; Supplementary Figure S2).

**Table 2:** Identification of BMD-associated risk genes from multiple omics datasets

Gene	LBF	Sherlock-identified *P* value (discovery dataset; corrected *P*<0.05)	Sherlock-identified *P* value (replication dataset; *P*<0.05)	MAGMA-identified *P* value (BMD GWAS; corrected *P*<0.05)	MAGMA-identified *P* value (Null GWAS; corrected *P*<0.05)
*MSRA*	10.59	7.87E-07	1.52E-06	5.29E-46	Non-significance
*BLK*	9.14	7.87E-07	9.26E-05	4.61E-35	Non-significance
*ACP2*	7.50	7.87E-07	7.59E-07	4.35E-14	Non-significance
*EPB41L2*	7.48	7.87E-07	7.59E-07	1.96E-18	Non-significance
*ACSS2*	6.84	7.87E-07	1.58E-03	6.81E-12	Non-significance
*ZFP57*	6.82	7.87E-07	7.59E-07	2.18E-06	Non-significance
*MARK3*	6.76	7.87E-07	1.82E-05	1.66E-37	Non-significance
*FDFT1*	6.66	7.87E-07	6.68E-05	5.00E-18	Non-significance
*PPP1CB*	6.62	7.87E-07	7.59E-07	4.01E-13	Non-significance
*SPTBN1*	6.61	7.87E-07	9.72E-05	9.60E-72	Non-significance
*AMT*	6.59	7.87E-07	7.59E-07	3.43E-11	Non-significance
*MPHOSPH9*	6.53	7.87E-07	8.81E-05	2.85E-09	Non-significance
*CPT1A*	6.49	7.87E-07	7.59E-07	1.92E-15	Non-significance
*XBP1*	6.43	7.87E-07	1.52E-06	1.49E-13	Non-significance
*CLDN23*	6.38	7.87E-07	1.67E-05	8.60E-23	Non-significance
*H1F0*	6.36	7.87E-07	4.28E-04	7.94E-08	Non-significance
*SSH2*	6.27	7.87E-07	9.11E-05	4.85E-16	Non-significance
*LACTB2*	6.27	7.87E-07	2.43E-05	2.19E-16	Non-significance
*VNN3*	6.25	7.87E-07	7.59E-07	8.52E-07	Non-significance
*DGKQ*	6.12	7.87E-07	6.83E-05	3.96E-16	Non-significance
*LAMB2*	6.08	7.87E-07	2.83E-02	6.99E-12	Non-significance
*TRIOBP*	5.96	7.87E-07	1.03E-04	2.42E-06	Non-significance
*TPCN2*	5.96	7.87E-07	1.52E-05	1.57E-21	Non-significance
*BAG5*	5.94	7.87E-07	1.52E-06	1.95E-25	Non-significance
*MRPL21*	5.84	1.57E-06	7.59E-07	1.67E-10	Non-significance
*CTSB*	5.79	1.57E-06	2.43E-05	8.22E-19	Non-significance
*NME4*	5.71	1.57E-06	2.58E-05	6.83E-13	Non-significance
*TRIP11*	5.70	1.57E-06	1.52E-06	1.11E-08	Non-significance
*PIGN*	5.55	1.57E-06	1.52E-06	2.99E-07	Non-significance
*ATXN3*	5.46	1.57E-06	4.56E-04	8.28E-09	Non-significance
*ST7L*	5.40	1.57E-06	7.59E-07	2.38E-17	Non-significance
*WARS2*	5.35	1.57E-06	1.52E-05	2.64E-11	Non-significance
*CLMN*	5.14	1.57E-06	2.38E-03	1.55E-08	Non-significance
*CKB*	5.14	1.57E-06	7.59E-07	9.61E-38	Non-significance
*UBE2E3*	5.06	1.57E-06	1.93E-04	2.85E-08	Non-significance
*CIB1*	5.06	1.57E-06	1.52E-06	5.56E-07	Non-significance

**Figure 3:**
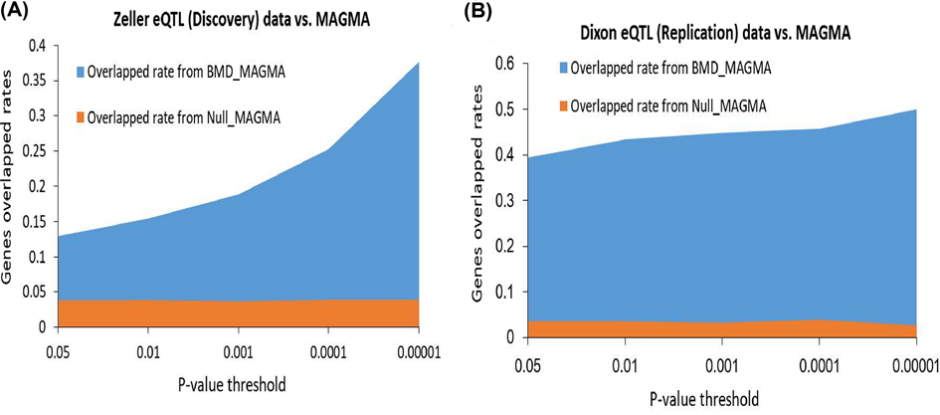
Genes identified from Sherlock Bayesian analysis validated by MAGMA-identified genes (**A**) Sherlock-identified genes from Zeller eQTL dataset (discovery stage) were higher overlapped with MAGMA-identified genes from BMD GWAS (Dataset #1) than that from Null-based GAWS (Dataset #2) at five different *P*-value thresholds of 0.05, 0.01, 0.001, 0.0001, and 0.00001. (**B**) Sherlock-identified genes from Dixon eQTL dataset (validation stage) were higher overlapped with MAGMA-identified genes from BMD GWAS (Dataset #1) than that from Null-based GAWS (Dataset #2) at five different *P*-value thresholds of 0.05, 0.01, 0.001, 0.0001, and 0.00001.

Furthermore, we found that no matter significant or suggestive genes identified in the *Sherlock* discovery stage were significantly higher overlapped with genes identified in the *Sherlock* validation stage and MAGMA validation (permutation *P* < 1.0 × 10^−5^; see Figure 2B,C and Supplementary Figure S3A,B). More interestingly, comparing the MAGMA results of Null GWAS, we observed that *Sherlock*-identified genes from the discovery and validation stage have significantly high overlap rates with MAGMA-identified genes from BMD GWAS than that from Null GWAS (Figure 3A,B).

### PPI analysis of 36 BMD-associated risk genes identified by Sherlock analysis

**Figure 4:**
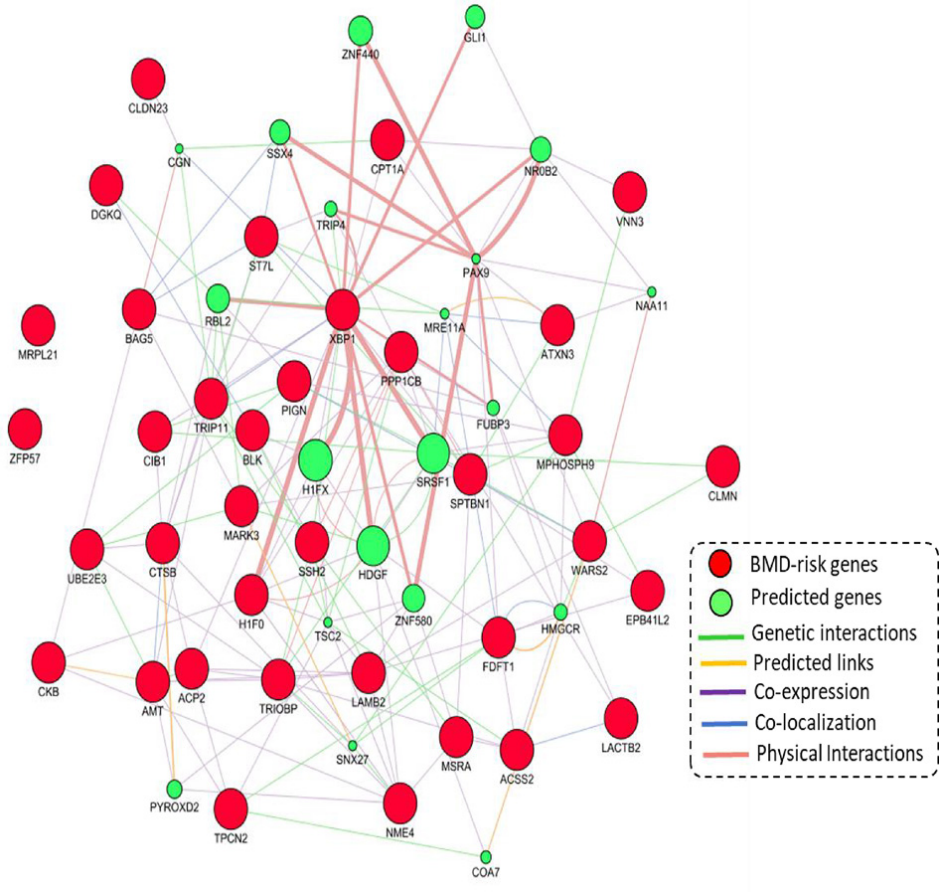
Protein–protein interactions between proteins encoded by 36 identified BMD-risk genes based on the application of the GeneMANIA database The 36 BMD-risk genes were provided as query (red nodes) and a number of additional genes were predicted to be linked (green nodes). The predicted attributes included co-localization, genetic interactions, predicted links, co-expression, and physical interactions.

To determine whether these 36 BMD-associated risk genes identified by *Sherlock* and MAGMA analysis interact with each other, we conducted a PPI network-based analysis using interactions of genetic interaction, prediction links, co-expression, co-localization, and physical interactions based on the GeneMANIA database [35]. Figure 4 shows that these BMD-associated risk genes are constructed a biological network, indicating there exist highly biological connections between these identified genes. For example, the hub genes of *PPP1CB, XBP1, FDFT1*, and *SPTBN1* have the most number of interactions with both predicted genes and BMD-associated genes (Figure 4). Additionally, the hub gene of *XBP1* shows strong evidence of physical interactions with BMD-associated genes of *H1F0* and predicted genes of *ZNF440, SRSF1*, and *H1FX* based on the interactions from BIOGRID and IREF databases [37,38]. The hub gene of *PPP1CB* has genetic interactions with BMD-associated genes of *SPTBN1, TRIOP*, and *SSH2* (Figure 4) based on a genome-wide human genetic interaction map [39].

### Differential expression analysis of identified genes in high BMD and low BMD subjects

**Figure 5:**
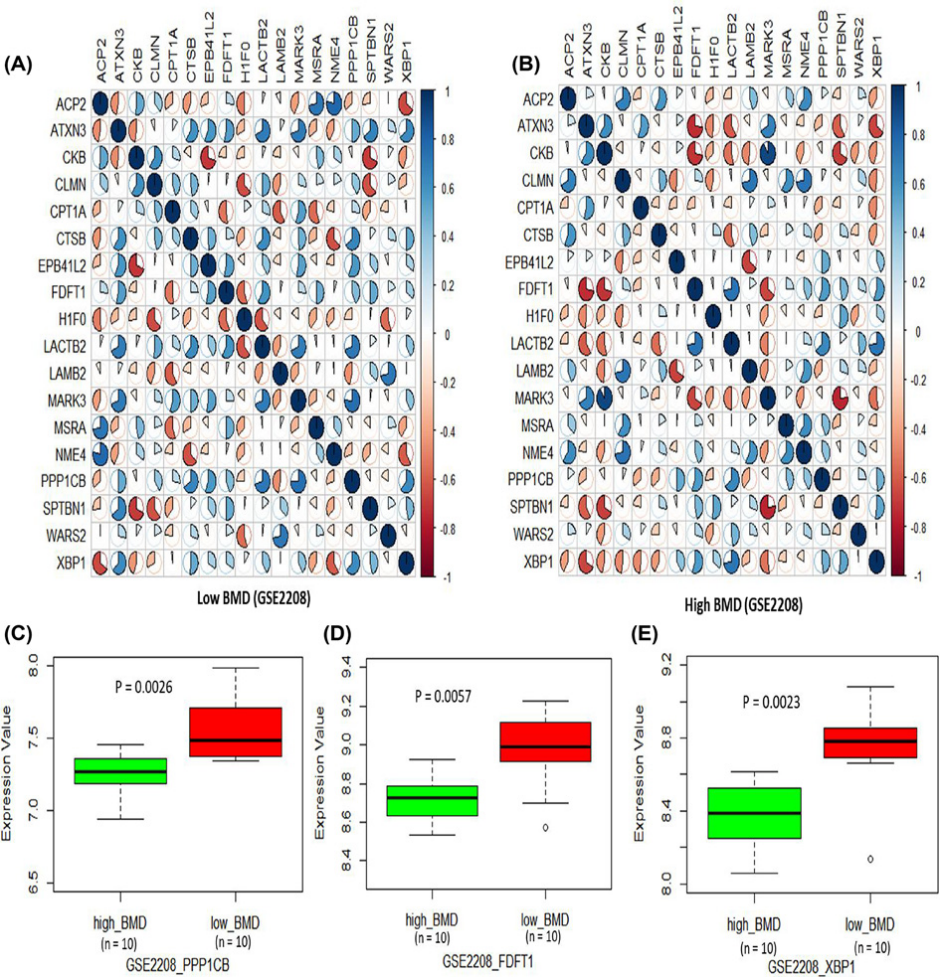
Differential expression profiles of 36 genes between high BMD and low BMD subjects in the GSE2208 dataset (**A**) Co-expression patterns of 36 risk genes in low BMD subjects in the GSE2208 dataset. (**B**) Co-expression patterns of 36 risk genes in high BMD subjects in the GSE2208 dataset. (**C**) Boxplot of the different gene expression of *PPP1CB* between high BMD and low BMD subjects in the GSE2208 dataset. (**D**) Boxplot of the different gene expression of *FDFT1* between high BMD and low BMD subjects in the GSE2208 dataset. (**E**) Boxplot of the different gene expression of *XBP1* between high BMD and low BMD subjects in the GSE2208 dataset.

**Figure 6:**
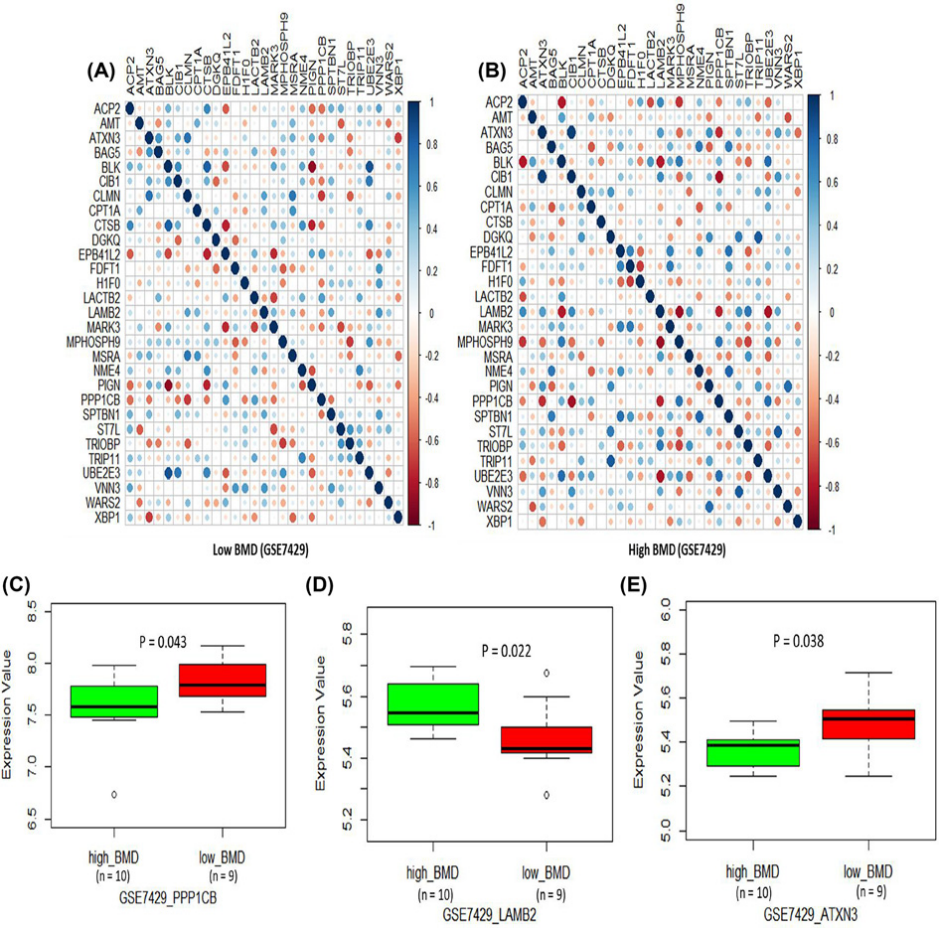
Differential expression profiles of 36 genes between high BMD and low BMD subjects in the GSE7429 dataset (**A**) Co-expression patterns of 36 risk genes in low BMD subjects in the GSE7429 dataset. (**B**) Co-expression patterns of 36 risk genes in high BMD subjects in the GSE7429 dataset. (**C**) Boxplot of the different gene expression of *PPP1CB* between high BMD and low BMD subjects in the GSE7429 dataset. (**D**) Boxplot of the different gene expression of *FDFT1* between high BMD and low BMD subjects in the GSE7429 dataset. (**E**) Boxplot of the different gene expression of *XBP1* between high BMD and low BMD subjects in the GSE7429 dataset.

Furthermore, we tested the co-expression patterns of these identified genes between high BMD and low BMD subjects based on two independent RNA expression datasets (i.e. GSE2208 and GSE7429). Based on the Pearson correlation analysis, we found that the co-expression patterns of these genes showed obvious differences between high and low BMD (Figures 5A,B and 6A,B). Subsequently, we first performed a differential gene expression (DGE) analysis to test the expression level of these genes in high BMD and low BMD subjects in the GSE2208 dataset. We found the genes of *PPP1CB* (*P* = 0.0026), *FDFT1* (*P* = 0.0057), and *XBP1* (*P* = 0.0023) showed significant up-regulated expression in low BMD subjects compared with high BMD subjects (Figure 5C–E). Consistently, we found the *PPP1CB* gene showed significantly higher expression in low BMD subjects than that in high BMD subjects with the use of an independent dataset of GSE7429 (*P* = 0.043; Figure 6C). In the dataset of GSE7429, we also detected that *LAMB2* (*P* = 0.022) and *ATXN3* (*P* = 0.038) were significantly differential expressed between low BMD and high BMD subjects (Figure 6D,E). In addition, several genes showed marginal evidence of differences between low BMD and high BMD subjects in both datasets (Supplementary Figures S4 and S5).

### Replication and refinement of eQTL and GWAS results for PPP1CB

Furthermore, we concentrated our analysis on the hub gene of *PPP1CB*, which is a hub gene in our PPI network analysis. There were two eSNPs of rs9309664 (*P*_eQTL_ = 1.42 × 10^−17^ and *P*_GWAS_ = 1.40 × 10^−11^; see Supplementary Table S2) and rs7475 (*P*_eQTL_ = 2.10 × 10^−6^ and *P*_GWAS_ = 1.70 × 10^−7^; see Supplementary Table S4) in *PPP1CB* identified. To validate our results, we used the tool of HaploReg [40] to examine the association of two eSNPs (rs9309664 and rs7475) with the expression of *PPP1CB* in independent datasets. The result confirmed that rs9309664 is significantly associated with *PPP1CB* expression in blood cells from two independent datasets (*P* = 6.19 × 10^−6^ and *P* = 3.4 × 10^−26^; Supplementary Table S5), as well as rs7475 showed significant association with *PPP1CB* expression in a glioblastoma cell line from an independent dataset (*P* = 0.00024; Supplementary Table S5).

## Discussion

Hitherto, more than 200 BMD-associated loci have been identified by GWAS studies [6–8,10]. However, the underlying biological mechanism of these risk SNPs and genes are still intractability.

Due to the influence of genetic LD among SNPs, these documented BMD-associated loci usually contain numerous highly LD SNPs with similar significant association signals. Thus, to confirm the exact causal SNPs of BMD-associated loci appeared to be a tough job. In view of a great number of detected susceptibility SNPs were mapped into the non-coding genomic regions, we reasonably inferred that these reported SNPs confer susceptibility to BMD via regulation of gene expression level. To address this issue, an effective and powerful inference method is warranted to be developed to combine the multi-omics information from genetic associations of existing GWAS summary data and independent eQTL data. In the current investigation, we employed a Bayesian integrative approach to combine the genetic associations from the large-scale GWAS of BMD (*N* = 142,487) and two independent eQTL datasets. In total, we identified 36 promising genes with eSNPs potentially implicated in BMD risk. To further prioritize the most convincing gene, we carried out several bioinformatics analyses, including MAGMA gene-based analysis, pathway-based enrichment analysis, *in silico* permutation analysis, PPI network-based analysis, and DGE analysis. Our results revealed that several genes, especially the *PPP1CB* gene, may represent authentic susceptibility genes for BMD.

The standard process of GWAS-based data analysis commonly uses millions of genetic SNPs to identify a number of common SNPs that showed significant associations with traits of interest such as osteoporosis. Nevertheless, the power of GWAS has been conspicuously limited by the enormous number of examined variants. GWAS may ignore the comprehensively integrated effects of modest variants or genes, which not reach a genome-wide significance but still importantly implicated in the pathology of osteoporosis. Furthermore, only based on the findings of GWAS analysis is impossible to infer whether the detected disease-associated SNPs contain regulatory functions. Thus, *Sherlock* integrative analysis is a good and effective method for combining the information of GWAS with eQTL data and has been applied to identify many novel risk genes of many complex diseases [15,19–22], which cannot be detected by GWAS alone.

With the use of *Sherlock* integrative analysis and MAGMA gene-based analysis, there were 36 BMD-associated risk genes identified with eSNPs at a genome-wide significance; for example, *ACP2* (cis-rs11039035, *P*_GWAS_ = 1.10 × 10^−11^; cis-rs2290148, *P*_GWAS_ = 2.40 × 10^−8^), *EPB4L2* (cis-rs9375797, *P*_GWAS_ = 1.70 × 10^−8^; cis-rs4897473, *P*_GWAS_ = 2.40 × 10^−15^), *VNN3* (cis-rs9402490, *P*_GWAS_ = 9.40 × 10^−67^; cis-rs1856293, *P*_GWAS_ = 2.30 × 10^−10^), and *CKB* (cis-rs12894275, *P*_GWAS_ = 7.20 × 10^−9^; cis-rs2071407, *P*_GWAS_ = 1.70 × 10^−12^). Growing evidence indicates that disease-related genes encode functional proteins, which commonly generate a high interaction network [14,33,34,41]. By conducting a PPI network-based analysis, we found that these 36 identified genes appeared to be highly connected to each other. There were several genes of *PPP1CB, XBP1, FDFT1*, and *SPTBN1* with the most number of interactions as the hub genes of the enriched network. For the hub gene of *PPP1CB*, there were two significant eSNPs of rs9309644 (*P*_eQTL_ = 1.42 × 10^−17^ and *P*_GWAS_ = 1.40 × 10^−11^) and rs7475 (*P*_eQTL_ = 2.10 × 10^−6^ and *P*_GWAS_ = 1.70 × 10^−7^) identified. Consistently, these two eSNPs were reported to be significantly associated with *PPP1CB* expression in the other three previous studies [42–44]. Furthermore, based on two independent expression datasets, we found *PPP1CB* showed significantly higher expression in the low BMD group than that in the high BMD group. These findings indicate that the *PPP1CB* gene plays an important role in low BMD subjects, which is a strong risk factor for osteoporosis and a key factor for its diagnosis and treatment [2].

The protein encoded by *PPP1CB* gene, which is located on the chromosome of 2p23.2, is one of the three catalytic subunits of protein phosphatase 1 (PP1). Phosphoprotein phosphatase 1 (PPP1), a major type 1 serine/threonine phosphatase, is ubiquitously expressed and regulates various cellular functions including glycogen metabolism, cell division, and muscle contractility [45–48]. In recent, a study [49] showed a role for *PPP1CB* that it is the myosin light chain phosphatase responsible for Ca^2+^-transient rise and enhanced cell shortening in cardiomyocytes. Additionally, Cho et al. [45] have reported that PPP1CB regulates adipocyte differentiation partially by targeting the transcription factor C/EBPδ. Protein phosphatases are pivotal enzymes in diverse eukaryotic physiological processes including cell differentiation and proliferation, cell–cell communication, and regulation of transmembrane and intracellular signaling pathways. Dysregulation of these proteins expression or activity has been documented to be linked with numerous human diseases [45,50,51], including cancer, obesity, and osteoporosis.

In sum, our present study highlights 36 convincing genes associated with BMD risk and further provides strong evidence to support that *PPP1CB* represents a genuine BMD-associated risk gene with eSNPs conferring susceptibility to osteoporosis. Through integrating the GWAS-based genetic associations with gene expression data, we demonstrated a plausible elucidation of the biological function of genetic variants on osteoporosis susceptibility. Current integrative genomics analysis offers an effective pipeline for incorporating SNPs across the whole genome to genes via cis- and/or trans-regulatory effects on transcriptional abundance, which is more powerful for interpreting the biological mechanism of functional SNPs than a pure GWAS method. Identification of risk genes from our results give a group of new molecular targets for subsequent functional experiments and therapeutic testing in future, which will improve the translation of pharmacogenomics testing into clinical practice. Furthermore, more related molecular studies are warranted to reveal the biological mechanism of *PPP1CB* gene implicated in osteoporosis.

## Supplementary Material

Supplementary Figures S1-S5 and Tables S1-S5

## Abbreviations

BMD: bone mineral density
DGE: differential gene expression
eQTL: expression quantitative trait loci
eSNP: expression-associated single-nucleotide polymorphism
GEFOS: the Genetic Factors for Osteoporosis
GHS: the Gutenberg Heart study
GO: Gene Ontology
GWAS: genome-wide association study
KEGG: Kyoto Encyclopedia of Genes and Genomes
LD: linkage disequilibrium
MAGMA: Multi-marker Analysis of GenoMic Annotation
PPI: protein–protein interaction
