# Supplementary material for: Integrative genomics analysis of eQTL and GWAS summary data identifies PPP1CB as a novel bone mineral density risk genes
Source: Biosci Rep. 2020 Apr 21;40(4):BSR20193185. doi: 10.1042/BSR20193185 (PMC7178214; doi:10.1042/BSR20193185)
Supplement: Supplementary Figures S1-S5 and Tables S1-S5 [file BSR-2019-3185_supp.pdf]

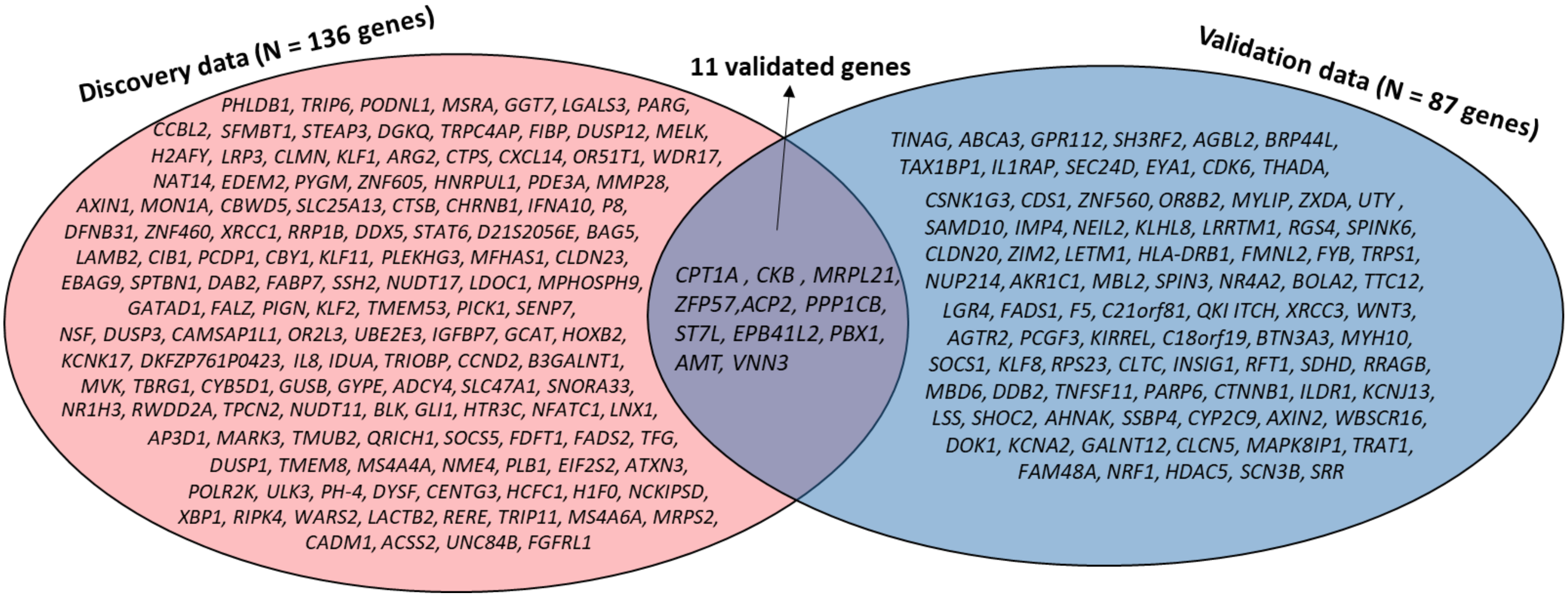

Supplemental Figure S1

Supplemental Figure S1. Venn plot of BMD-associated genes identified by Sherlock Bayesian analysis based on two independent datasets. There were 11 validated genes conferring risk to BMD. The P values of all genes were corrected by the method of Bonferroni correction.

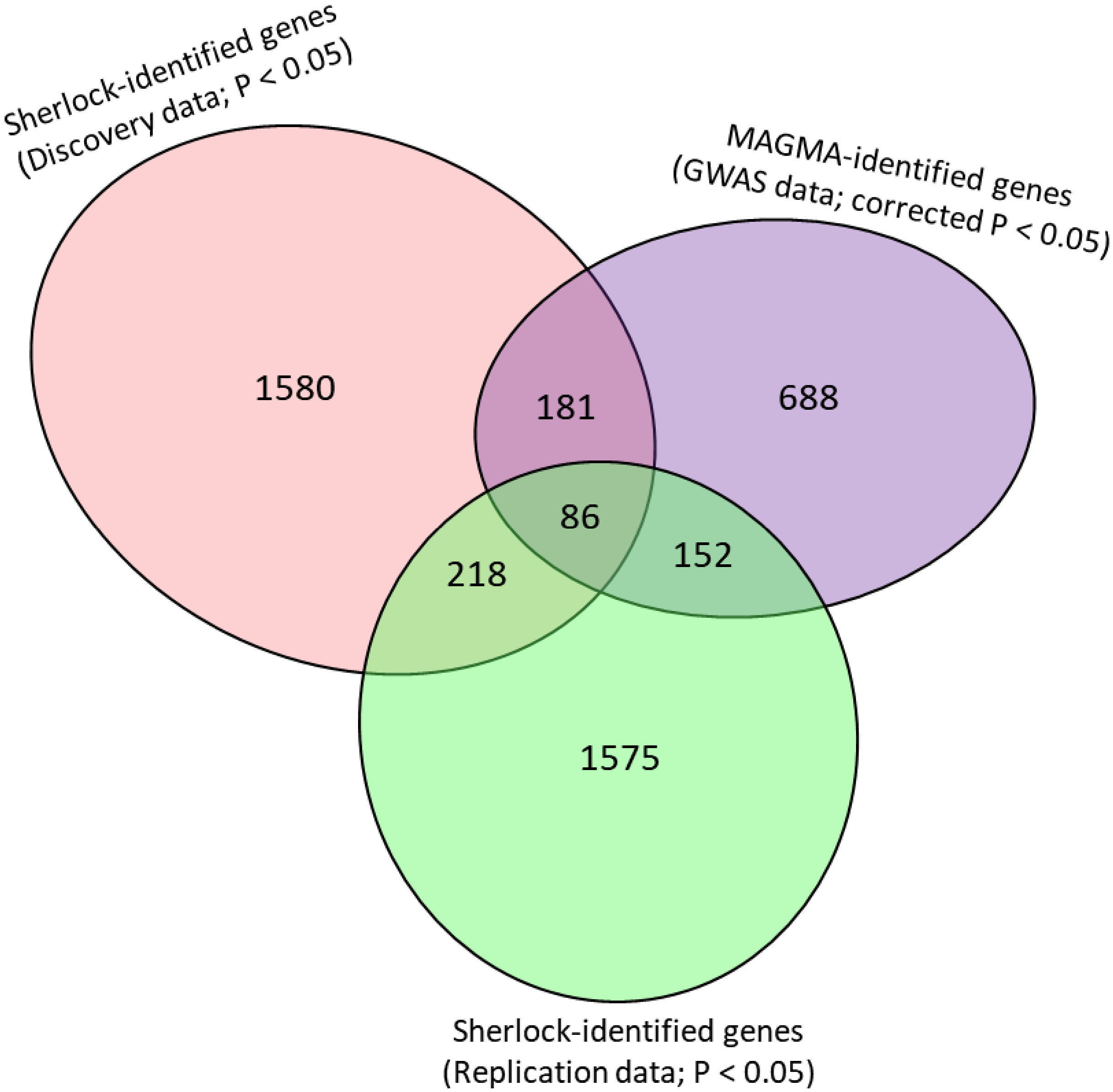

Supplemental Figure S2

Supplemental Figure S2. Venn plot of BMD-relevant genes based on three datasets: Sherlock-identified genes from the discovery dataset (P < 0.05); Sherlock-identified genes from the validation dataset (P < 0.05); MAGMA-identified genes from the GWAS dataset (corrected P < 0.05).

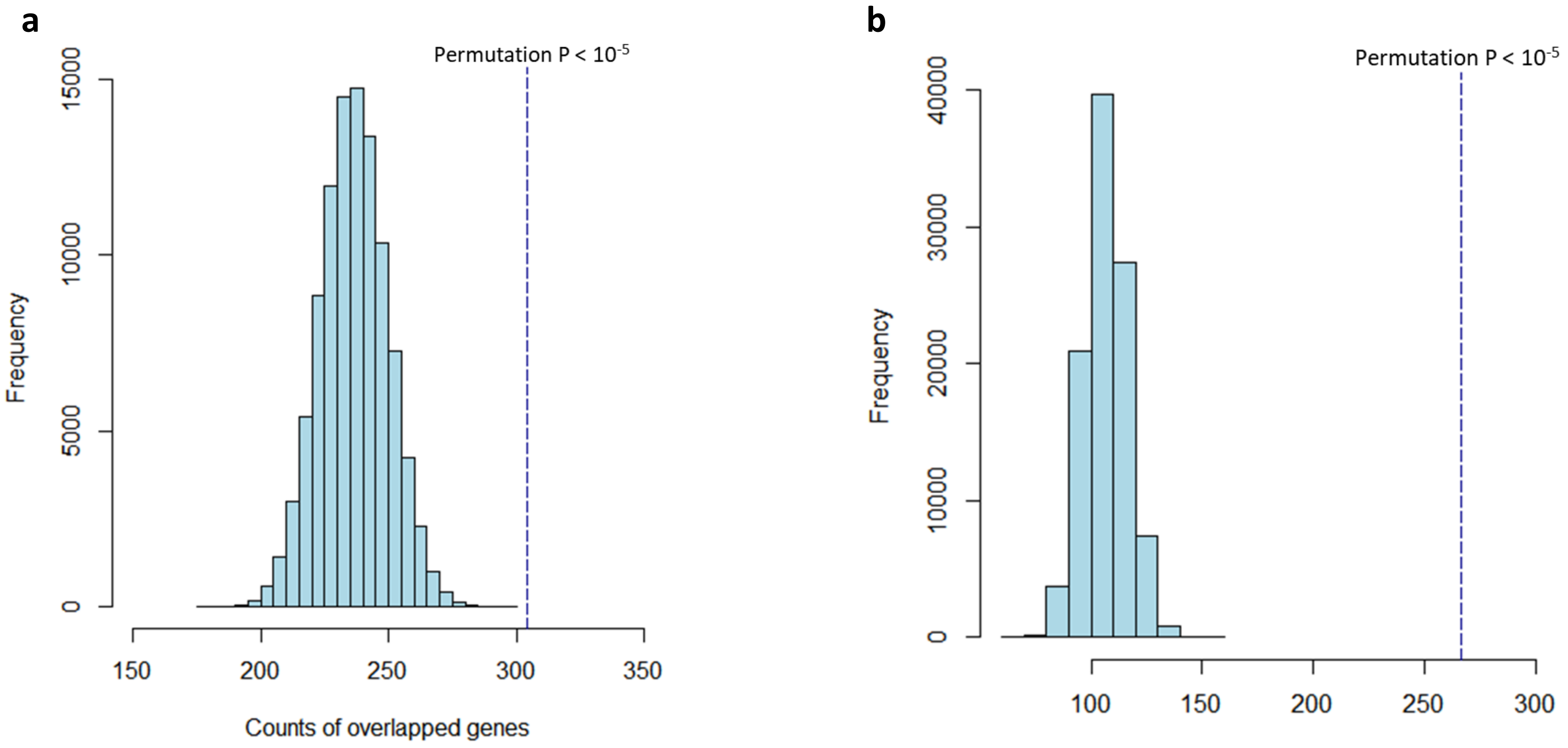

Zeller eQTL (Discovery) data vs. Dixon eQTL (Replication)

Zeller eQTL (Discovery) data vs. MAGMA

Supplemental Figure S3

Supplemental Figure S3. Computer-based permutation analysis. a) Computer-based permutation analysis (100,000 times) of the counts of risk genes from Zeller eQTL dataset ($P < 0.05$; discovery stage) overlapped with that from Dixon eQTL dataset ($P < 0.05$; Replication stage). b) Computer-based permutation analysis (100,000 times) of the counts of risk genes from Zeller eQTL dataset ($P < 0.05$; discovery stage) overlapped with that from MAGMA-based dataset (corrected $P < 0.05$; technical validation stage).

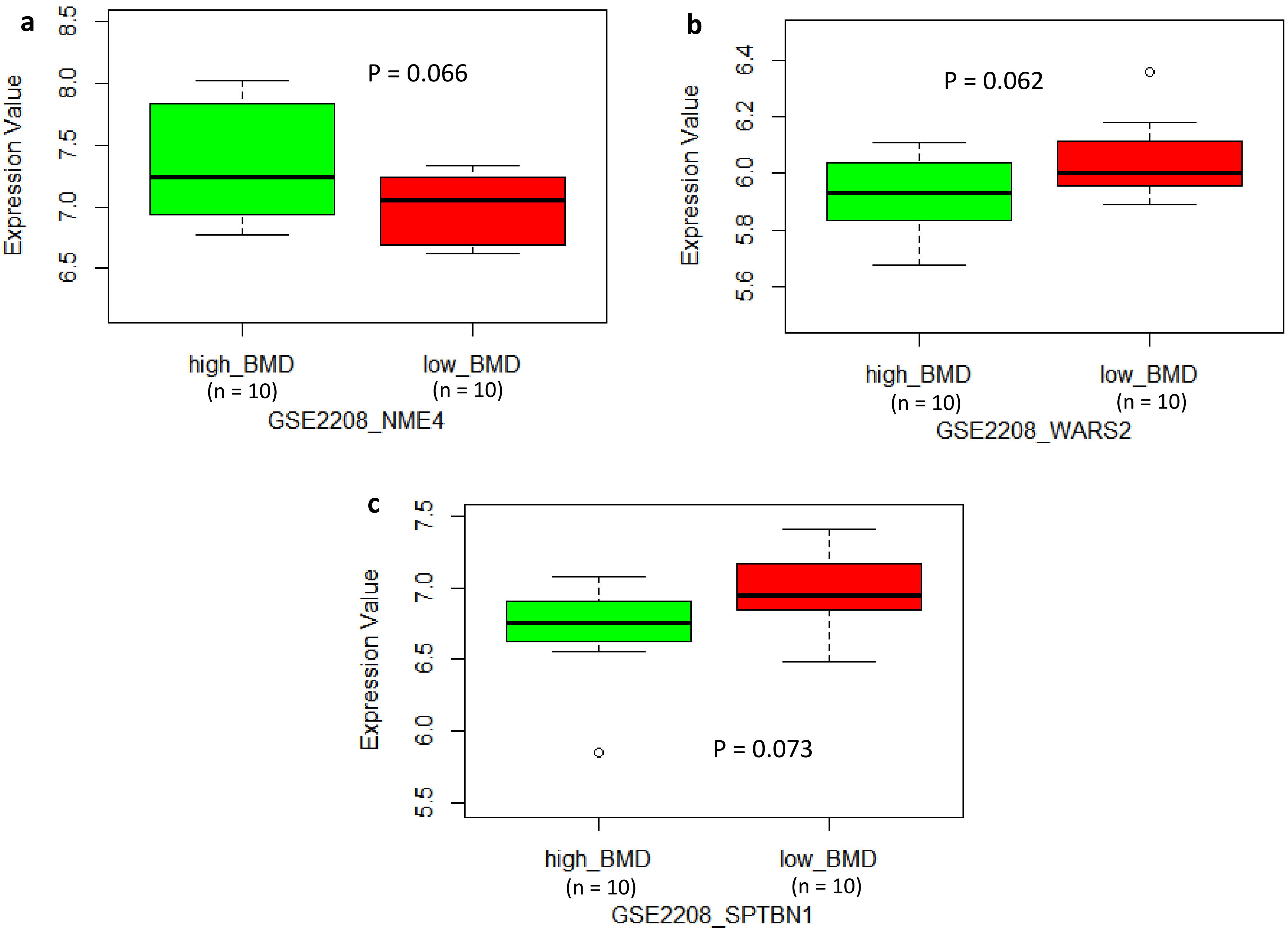

Supplemental Figure S4

Supplemental Figure S4. Boxplots show the expression profile of identified genes between high BMD and low BMD in GSE2208 dataset. a) Boxplot of the different gene expression of *NME4*between high BMD and low BMD subjects in GSE2208 dataset. b) Boxplot of the different gene expression of *WARS2*between high BMD and low BMD subjects in GSE2208 dataset. c) Boxplot of the different gene expression of *SPTBN1*between high BMD and low BMD subjects in GSE2208 dataset.

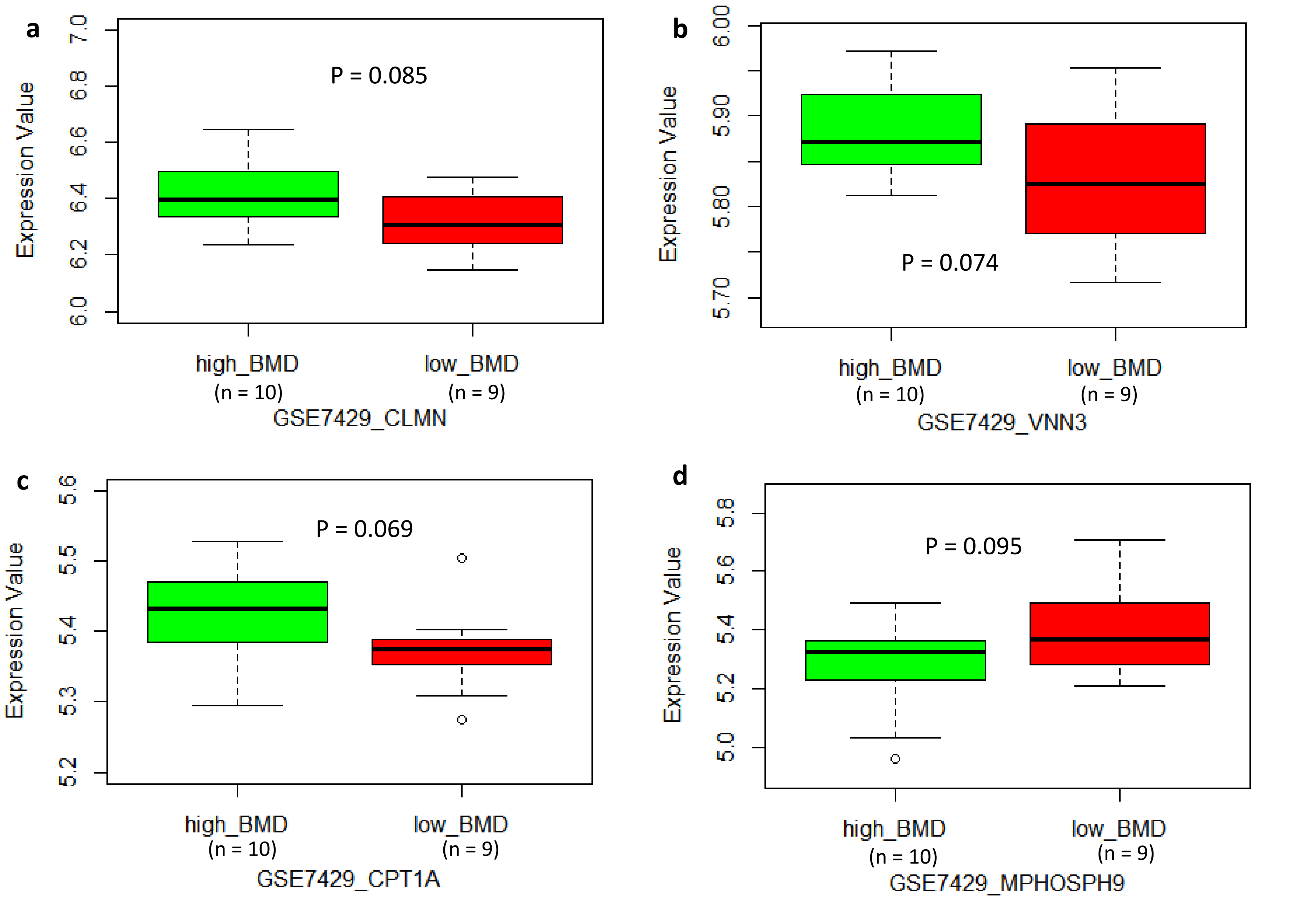

Supplemental Figure S5

Supplemental Figure S5. Boxplots show the expression profile of identified genes between high BMD and low BMD in GSE2208 dataset. a) Boxplot of the different gene expression of*CLMN*between high BMD and low BMD subjects in GSE7429 dataset. b) Boxplot of the different gene expression of *VNN3*between high BMD and low BMD subjects in GSE7429 dataset. c) Boxplot of the different gene expression of *CPT1A*between high BMD and low BMD subjects in GSE7429 dataset. d) Boxplot of the different gene expression of*MPHOSPH9*between high BMD and low BMD subjects in GSE7429 dataset.

Supplemental Table S1. ***Sherlock* integrative analysis identifies 147 genes as BMD risk genes in the discovery stage**

Gene	LBF	Gene P value
NR1H3	12.2999	7.87E-07
MVK	11.5158	7.87E-07
TBRG1	11.5094	7.87E-07
PDE3A	11.315	7.87E-07
FALZ	11.1695	7.87E-07
EBAG9	10.8478	7.87E-07
MSRA	10.5948	7.87E-07
ULK3	9.99511	7.87E-07
HOXB2	9.76989	7.87E-07
GATAD1	9.5143	7.87E-07
BLK	9.14053	7.87E-07
MS4A4A	9.04221	7.87E-07
LDOC1	9.01994	7.87E-07
P8	8.71756	7.87E-07
WDR17	8.64742	7.87E-07
NAT14	8.58634	7.87E-07
MFHAS1	8.57354	7.87E-07
TMEM53	8.52609	7.87E-07
ADCY4	8.49684	7.87E-07
SENP7	8.47952	7.87E-07
RIPK4	8.19958	7.87E-07
PLEKHG3	7.91651	7.87E-07
PICK1	7.78789	7.87E-07
PARG	7.53614	7.87E-07
ACP2	7.49565	7.87E-07
GCAT	7.49433	7.87E-07
EPB41L2	7.47978	7.87E-07
CXCL14	7.30761	7.87E-07
PODNL1	7.22259	7.87E-07
GUSB	7.11892	7.87E-07
PH-4	7.10204	7.87E-07
CBY1	7.05232	7.87E-07
CBWD5	6.9474	7.87E-07
UNC84B	6.90261	7.87E-07
TMUB2	6.88889	7.87E-07
RERE	6.88072	7.87E-07
DUSP1	6.84233	7.87E-07
ACSS2	6.83981	7.87E-07
ZFP57	6.8172	7.87E-07
HNRPUL1	6.78973	7.87E-07
EDEM2	6.78454	7.87E-07
MARK3	6.75701	7.87E-07
PBX1	6.68498	7.87E-07
FDFT1	6.65806	7.87E-07
CADM1	6.64867	7.87E-07
FABP7	6.64681	7.87E-07
AP3D1	6.6278	7.87E-07
PPP1CB	6.61774	7.87E-07
SPTBN1	6.61328	7.87E-07
AMT	6.59051	7.87E-07

DUSP12	6.5719	7.87E-07
LRP3	6.57174	7.87E-07
PLB1	6.55676	7.87E-07
ARG2	6.53133	7.87E-07
MPHOSPH9	6.53093	7.87E-07
STEAP3	6.5064	7.87E-07
CPT1A	6.49275	7.87E-07
XBP1	6.43078	7.87E-07
CCBL2	6.42554	7.87E-07
TRIP6	6.42465	7.87E-07
CLDN23	6.38306	7.87E-07
AXIN1	6.38213	7.87E-07
H1F0	6.35558	7.87E-07
DAB2	6.316	7.87E-07
SLC47A1	6.29566	7.87E-07
FGFRL1	6.29175	7.87E-07
SOCS5	6.2903	7.87E-07
OR2L3	6.28478	7.87E-07
SSH2	6.27166	7.87E-07
LACTB2	6.26756	7.87E-07
KLF11	6.25736	7.87E-07
MS4A6A	6.25532	7.87E-07
VNN3	6.24743	7.87E-07
B3GALNT1	6.20561	7.87E-07
NFATC1	6.20172	7.87464E-07
MRPS2	6.1761	7.87E-07
RWDD2A	6.15794	7.87E-07
FIBP	6.13312	7.87E-07
CYB5D1	6.12814	7.87E-07
DGKQ	6.12386	7.87E-07
KLF1	6.11453	7.87E-07
LAMB2	6.07699	7.87E-07
IL8	5.99556	7.87E-07
OR51T1	5.99219	7.87E-07
TRIOBP	5.95978	7.87E-07
NSF	5.95953	7.87E-07
TPCN2	5.95545	7.87E-07
TMEM8	5.95372	7.87E-07
QRICH1	5.94704	7.87E-07
BAG5	5.9431	7.87E-07
STAT6	5.8953	7.87E-07
CAMSAP1L1	5.88891	7.87E-07
MON1A	5.87306	1.57E-06
MRPL21	5.83995	1.57E-06
CTSB	5.78827	1.57E-06
TRPC4AP	5.78065	1.57E-06
DDX5	5.76447	1.57E-06
GGT7	5.71233	1.57E-06
SNORA33	5.71046	1.57E-06
KLF2	5.71025	1.57E-06
NME4	5.70779	1.57E-06
TRIP11	5.70165	1.57E-06
GLI1	5.69007	1.57E-06
IFNA10	5.68755	1.57E-06
IDUA	5.62864	1.57E-06

EIF2S2	5.62145	1.57E-06
MMP28	5.62022	1.57E-06
ZNF605	5.61031	1.57E-06
SLC25A13	5.58744	1.57E-06
PHLDB1	5.58561	1.57E-06
DUSP3	5.5704	1.57E-06
DFNB31	5.56622	1.57E-06
PIGN	5.55191	1.57E-06
CTPS	5.48714	1.57E-06
LNX1	5.48174	1.57E-06
ATXN3	5.45739	1.57E-06
NCKIPSD	5.44234	1.57E-06
GYPE	5.41442	1.57E-06
ST7L	5.40094	1.57E-06
NUDT17	5.3943	1.57E-06
WARS2	5.35449	1.57E-06
POLR2K	5.33098	1.57E-06
DYSF	5.29734	1.57E-06
PCDP1	5.29729	1.57E-06
CHRNB1	5.27137	1.57E-06
NUDT11	5.26999	1.57E-06
ZNF460	5.26737	1.57E-06
FADS2	5.26155	1.57E-06
PYGM	5.24992	1.57E-06
HTR3C	5.23737	1.57E-06
SFMBT1	5.22316	1.57E-06
D21S2056E	5.22062	1.57E-06
LGALS3	5.21765	1.57E-06
MELK	5.19724	1.57E-06
TFG	5.18289	1.57E-06
RRP1B	5.1736	1.57E-06
CLMN	5.14477	1.57E-06
CKB	5.14417	1.57E-06
CENTG3	5.11152	1.57E-06
KCNK17	5.08362	1.57E-06
IGFBP7	5.08341	1.57E-06
XRCC1	5.0827	1.57E-06
UBE2E3	5.0613	1.57E-06
CIB1	5.05829	1.57E-06
H2AFY	5.04571	1.57E-06
CCND2	5.03881	1.57E-06
HCFC1	5.02937	1.57E-06

Supplemental Table S2. The results of 11 BMD-associated genes in the discovery stage

Gene name	LBF[a]	P value[b]	Cis or trans SNP[c]	P value from eQTL[d]	P value from GWAS[e]	Correction-based P value[f]
ACP2	7.50	7.87×10^{-7}	rs11039035 (cis)	3.71×10^{-12}	1.10×10^{-11}	< 0.05
EPB41L2	7.48	7.87×10^{-7}	rs9375797 (cis)	3.47×10^{-8}	1.70×10^{-8}	< 0.05
ZFP57	6.82	7.87×10^{-7}	rs3129063 (cis)	1.46×10^{-29}	4.50×10^{-6}	< 0.05
PBX1	6.68	7.87×10^{-7}	rs4581300 (trans)	1.57×10^{-6}	2.70×10^{-7}	< 0.05
PPP1CB	6.62	7.87×10^{-7}	rs9309664 (cis)	1.42×10^{-17}	1.40×10^{-11}	< 0.05
AMT	6.59	7.87×10^{-7}	rs3749237 (cis)	2.48×10^{-10}	4.60×10^{-11}	< 0.05
CPT1A	6.49	7.87×10^{-7}	rs3736228 (cis)	3.93×10^{-17}	6.50×10^{-19}	< 0.05
VNN3	6.25	7.87×10^{-7}	rs9402490 (cis)	5.76×10^{-12}	9.40×10^{-67}	< 0.05
MRPL21	5.84	1.57×10^{-6}	rs646586 (cis)	1.37×10^{-86}	8.40×10^{-9}	< 0.05
ST7L	5.40	1.57×10^{-6}	rs3790608 (cis)	8.16×10^{-22}	1.20×10^{-18}	< 0.05
CKB	5.14	1.57×10^{-6}	rs12894275 (cis)	2.14×10^{-8}	7.20×10^{-9}	< 0.05

Note: [a] LBF:The abbreviation of the logarithm of the Bayes factor. LBF considers as a vital indicator to evaluate the association of a risk gene and complicate disease such as BMD in current study via pooling the eQTL data and GWAS summary statistics. The large value of LBF highly reflects the high links between the gene and BMD; e.g., a number of 7.5 LBF value (*exp*(7.50) = 1,808 times) represents the gene of interest preferably shows prominent association with BMD than non-significant association underlying the null hypothesis.

[b]P–value from calculation based on the statistical inferences method of *Sherlock* integrative analysis. Considering *Sherlock* employs finite times of permutation test to calculate the P value for each gene, some of these top-ranked genes (e.g., *ACP2* and *EPB41L2*) have distinct LBF values but

obtain the same rankings (namely, their P values are same).

[c]We only showed the SNP has the highest score of LBF in each gene.

[d]P-value from expression quantitative trait analysis of Zeller et al.

[e]P-value from GWAS on BMD of Kemp et al.

[f]The method of Bonferroni correction was employed to correct the P-values from *Sherlock* tool.

Supplemental Table S3. ***Sherlock*** **integrative analysis identifies 98 genes as BMD risk genes in the validation stage**

Gene	LBF	Gene P value
AMT	13.5691	7.59E-07
MAPK8IP1	11.7318	7.59E-07
SPIN3	11.5091	7.59E-07
MYLIP	10.9087	7.59E-07
MYH10	9.95042	7.59E-07
TTC12	9.80843	7.59E-07
SSBP4	9.76063	7.59E-07
BOLA2	9.50038	7.59E-07
NR4A2	9.29855	7.59E-07
ZFP57	8.9893	7.59E-07
NUP214	8.60844	7.59E-07
AGTR2	8.41611	7.59E-07
PBX1	8.26044	7.59E-07
ILDR1	7.97657	7.59E-07
IMP4	7.96895	7.59E-07
OR8B2	7.94505	7.59E-07
WNT3	7.85747	7.59E-07
EYA1	7.77769	7.59E-07
KCNJ13	7.51417	7.59E-07
LSS	7.4792	7.59E-07
CDK6	7.42466	7.59E-07
CDS1	7.4131	7.59E-07
RGS4	7.30141	7.59E-07
F5	7.20892	7.59E-07
DOK1	7.19648	7.59E-07
FADS1	7.16901	7.59E-07
SDHD	7.11542	7.59E-07
FMNL2	7.04845	7.59E-07
ITCH	6.84407	7.59E-07
MRPL21	6.82275	7.59E-07
HDAC5	6.81755	7.59E-07
C18orf19	6.79965	7.59E-07
CSNK1G3	6.64201	7.59E-07
NRF1	6.59861	7.59E-07
CLDN20	6.57949	7.59E-07
SH3RF2	6.43827	7.59E-07
CLCN5	6.42869	7.59E-07
WBSCR16	6.35304	7.59E-07
KLF8	6.31301	7.59E-07
MBL2	6.30824	7.59E-07
KIRREL	6.28072	7.59E-07
THADA	6.27297	7.59E-07
CKB	6.26649	7.59E-07
SCN3B	6.16817	7.59E-07
UTY	6.15071	7.59E-07
TNFSF11	6.14619	7.59E-07
AKR1C1	6.14427	7.59E-07

KLHL8	6.12499	7.59E-07
ZIM2	6.10742	7.59E-07
SHOC2	6.10708	7.59E-07
XRCC3	6.07382	7.59E-07
LRRTM1	6.04204	7.59E-07
AGBL2	5.9564	7.59E-07
LETM1	5.94412	7.59E-07
SRR	5.92776	7.59E-07
VNN3	5.92444	7.59E-07
EPB41L2	5.9032	7.59E-07
PPP1CB	5.8688	7.59E-07
SEC24D	5.86731	7.59E-07
TAX1BP1	5.84235	7.59E-07
SOCS1	5.8379	7.59E-07
RRAGB	5.7971	7.59E-07
RPS23	5.79692	7.59E-07
CTNNB1	5.77722	7.59E-07
SAMD10	5.6892	7.59E-07
QKI	5.66379	7.59E-07
PARP6	5.65955	7.59E-07
IL1RAP	5.58381	7.59E-07
ACP2	5.57894	7.59E-07
GALNT12	5.50698	7.59E-07
MBD6	5.49491	7.59E-07
KCNA2	5.46585	7.59E-07
ABCA3	5.45385	7.59E-07
PCGF3	5.37444	7.59E-07
ZNF560	5.37433	7.59E-07
FAM48A	5.37108	7.59E-07
CPT1A	5.35921	7.59E-07
ST7L	5.35852	7.59E-07
SPINK6	5.35312	7.59E-07
ZXDA	5.34714	7.59E-07
AXIN2	5.34304	7.59E-07
AHNAK	5.32541	7.59E-07
TRPS1	5.29976	7.59E-07
NEIL2	5.28915	7.59E-07
FYB	5.24885	7.59E-07
RFT1	5.23427	7.59E-07
BRP44L	5.20072	7.59E-07
CYP2C9	5.19215	7.59E-07
INSIG1	5.19194	7.59E-07
DDB2	5.15843	7.59E-07
GPR112	5.14337	7.59E-07
C21orf81	5.13445	7.59E-07
CLTC	5.07912	7.59E-07
BTN3A3	5.07595	7.59E-07
LGR4	5.05598	7.59E-07
HLA-DRB1	5.02535	1.52E-06
TINAG	5.01643	1.52E-06
TRAT1	5.0079	1.52E-06

Supplemental Table S4. The results of 11 BMD-associated genes in the validation stage

Gene name	LBF[a]	P value[b]	Cis or trans SNP[c]	P value from eQTL[d]	P value from GWAS[e]	Correction-based P value[f]
ACP2	5.58	7.59×10^{-7}	rs2290148 (cis)	5.00×10^{-5}	2.40×10^{-8}	< 0.05
EPB41L2	5.90	7.59×10^{-7}	rs4897473 (cis)	2.70×10^{-6}	2.40×10^{-15}	< 0.05
ZFP57	8.99	7.59×10^{-7}	rs2747430 (cis)	5.10×10^{-29}	5.30×10^{-7}	< 0.05
PBX1	8.26	7.59×10^{-7}	rs1487628 (trans)	2.3×10^{-6}	1.20×10^{-7}	< 0.05
PPP1CB	5.87	7.59×10^{-7}	rs7475 (cis)	2.10×10^{-6}	1.70×10^{-7}	< 0.05
AMT	13.57	7.59×10^{-7}	rs1464569 (cis)	6.70×10^{-6}	8.20×10^{-7}	< 0.05
CPT1A	5.36	7.59×10^{-7}	rs2156464 (cis)	6.60×10^{-8}	2.50×10^{-10}	< 0.05
VNN3	5.92	7.59×10^{-7}	rs1856293 (cis)	4.00×10^{-5}	2.30×10^{-10}	< 0.05
MRPL21	6.82	7.59×10^{-7}	rs629426 (cis)	2.80×10^{-9}	4.40×10^{-8}	< 0.05
ST7L	5.36	7.59×10^{-7}	rs1106287 (cis)	3.80×10^{-7}	4.70×10^{-15}	< 0.05
CKB	6.27	7.59×10^{-7}	rs2071407 (cis)	9.00×10^{-7}	1.70×10^{-12}	< 0.05

Note: [a] LBF:The abbreviation of the logarithm of the Bayes factor. LBF considers as a vital indicator to evaluate the association of a risk gene and complicate disease such as BMD in current study via pooling the eQTL data and GWAS summary statistics. The large value of LBF highly reflects the high links between the gene and BMD; e.g., a number of 5.58 LBF value (*exp*(5.58) = 265 times) represents the gene of interest preferably shows prominent association with BMD than non-significant association underlying the null hypothesis.

[b]P–value from calculation based on the statistical inferences method of *Sherlock* integrative analysis. Considering *Sherlock* employs finite times of permutation test to calculate the P value for each gene, some of these top-ranked genes (e.g., *ACP2* and *EPB41L2*) have distinct LBF values but obtain the same rankings (namely, their P values are same).

[c]We only showed the SNP has the highest score of LBF in each gene.

[d]P-value from expression quantitative trait analysis of Dixon et al.

[e]P-value from GWAS on BMD of Kemp et al.

[f]The method of Bonferroni correction was employed to correct the P-values from *Sherlock* tool.

Supplemental Table S5. **Additional evidence of eQTL findings for *PPP1CB* gene**

rs# of eSNP	Traits	P values	References (PMID)
rs9309664	Gene expression of SPDY1 in blood cells in Celiac disease	6.19×10^{-6}	[1](19128478)
rs9309664	Gene expression of PPP1CB in blood	3.4×10^{-26}	[2](21829388)
rs7475	Allele-specific Expression Patterns in human glioblastoma cell line U87MG	0.00024	[3](22467206)

References:

1. Heap, G.A., et al., *Complex nature of SNP genotype effects on gene expression in primary human leucocytes.* BMC Med Genomics, 2009. **2**: p. 1.
2. Fehrmann, R.S., et al., *Trans-eQTLs reveal that independent genetic variants associated with a complex phenotype converge on intermediate genes, with a major role for the HLA.* PLoS Genet, 2011. **7**(8): p. e1002197.
3. Li, G., et al., *Identification of allele-specific alternative mRNA processing via transcriptome sequencing.* Nucleic Acids Res, 2012. **40**(13): p. e104.
